# Effects of High Intensity Interval versus Moderate Continuous Training on Markers of Ventilatory and Cardiac Efficiency in Coronary Heart Disease Patients

**DOI:** 10.1155/2015/192479

**Published:** 2015-02-09

**Authors:** Gustavo G. Cardozo, Ricardo B. Oliveira, Paulo T. V. Farinatti

**Affiliations:** ^1^Amil Total Care, 22270-000 Rio de Janeiro, RJ, Brazil; ^2^Physical Activity and Health Promotion Laboratory of Rio de Janeiro State University (LABSAU), 20550-900 Rio de Janeiro, RJ, Brazil; ^3^Universidade Salgado de Oliveira (UNIVERSO), 24030-60 Niteroi, RJ, Brazil

## Abstract

*Background*. We tested the hypothesis that high intensity interval training (HIIT) would be more effective than moderate intensity continuous training (MIT) to improve newly emerged markers of cardiorespiratory fitness in coronary heart disease (CHD) patients, as the relationship between ventilation and carbon dioxide production (VE/VCO_2_ slope), oxygen uptake efficiency slope (OUES), and oxygen pulse (O_2_P). *Methods*. Seventy-one patients with optimized treatment were randomly assigned into HIIT (*n* = 23, age = 56 ± 12 years), MIT (*n* = 24, age = 62 ± 12 years), or nonexercise control group (CG) (*n* = 24, age = 64 ± 12 years). MIT performed 30 min of continuous aerobic exercise at 70–75% of maximal heart rate (HRmax), and HIIT performed 30 min sessions split in 2 min alternate bouts at 60%/90% HRmax (3 times/week for 16 weeks). *Results*. No differences among groups (before versus after) were found for VE/VCO_2_ slope or OUES (*P* > 0.05). After training the O_2_P slope increased in HIIT (22%, *P* < 0.05) but not in MIT (2%, *P* > 0.05), while decreased in CG (−20%, *P* < 0.05) becoming lower versus HIIT (*P* = 0.03). *Conclusion*. HIIT was more effective than MIT for improving O_2_P slope in CHD patients, while VE/VCO_2_ slope and OUES were similarly improved by aerobic training regimens versus controls.

## 1. Introduction

Cardiopulmonary exercise testing (CPX) has become increasingly applied in clinical practice because of its ability to noninvasively identify unexplained exercise intolerance, supporting decisions with regard to therapeutic interventions and helping prognosis estimate [1]. Among the ventilatory expired gas variables obtained during exercise testing, maximal oxygen consumption (peak VO_2_) remains the most frequently applied in both research and clinical settings.

However, despite the well-established value of peak VO_2_, the clinical role of other CPX variables such as the slope of the relationship between ventilation and carbon dioxide production (VE/VCO_2_ slope), oxygen uptake efficiency slope (OUES), and oxygen pulse (O_2_P) has emerged as valuable in clinical research. The VE/VCO_2_ slope and OUES have been shown to have complementary or even better survival prognostic value than peak VO_2_ in cardiac patients [2], while a flattening in O_2_P has been considered as a marker of ischemia [3].

In addition, indices of ventilatory exchange during exercise have become recognized as clinical markers of cardiac disease [1, 4, 5], as well as of improvement of cardiovascular function in cardiac patients [5, 6]. More recently, few studies have focused on the effects of training on markers of ventilatory and cardiac efficiency, such as VE/VCO_2_ slope, OUES, and O_2_P [7–10]. Although training has generally been shown to improve indices of ventilatory efficiency in heart failure patients, the results from these studies are somewhat mixed when CHD patients are considered [9, 10].

High intensity interval training (HIIT) has been shown to increase aerobic fitness more effectively than continuous moderate intensity training (MIT), therefore suggesting that it would confer greater cardioprotective benefits [2, 11–14]. Previous studies have investigated the effects of aerobic training with different intensities, applied to individuals with CHD [13, 15, 16]. In addition, a recent meta-analysis was published showing that HIIT appears to be more effective than continuous training in CHD patients [17].

We could find just one study demonstrating that different methods of aerobic training would be capable to improve the O_2_P peak in CHD patients [13]. Moreover, to date only one study examined the morphology of O_2_P curve of CHD at different times [18]. Certain physical training models should be tested in CHD and seek improvements in clinical relevant variables.

However, despite the fact that observational studies have explored the clinical relevance of VE/VCO_2_ slope, OUES, and O_2_P, few studies have tested the influence of regular exercise training on their responses [19, 20]. Furthermore, comparisons between chronic HIIT and MIT on these markers are still poorly described in coronary heart disease (CHD) patients.

Thus, this randomized controlled trial aimed to compare the effects of 16 weeks of HIIT and MIT and without exercise upon peak VO_2_, VE/VCO_2_ slope, OUES, and O_2_P in CHD patients. We tested the hypothesis that both HIIT and MIT would improve these markers versus controls in patients with CHD. It has been also hypothesized that gains induced by HIIT would be greater over MIT.

## 2. Methods

### 2.1. Subjects

Ninety-two patients referred to participate in a cardiac rehabilitation program participated in the study. Inclusion criteria were (a) history of coronary artery disease diagnosed by American Heart Association standard criteria [21]; (b) at least 35 years old; (c) ejection fraction (EF) greater than 50%. The following exclusion criteria were adopted: (a) recent acute myocardial infarction or revascularization (<3 months); (b) use of pacemaker; (c) musculoskeletal limitations that might affect participation in physical training or CPX; (d) attendance to less than 75% of the programmed training sessions or absence in four or more consecutive training sessions; (e) changes in medication classes and/or dosages during the study.

The study was carried out from January 2010 to January 2012. All patients signed an informed consent before enrolling in the study and the experimental protocol was approved by Institutional Ethics Committee.

### 2.2. Experimental Design

In the first visit to the laboratory those who attended all inclusion and exclusion criteria were identified and drug classes in use were recorded. In addition, measurements of weight and height, symptom-limited ramp CPX, and echocardiography at baseline were performed. From the 92 patients that underwent initial screening, 71 were considered eligible to participate in the study. In the second visit, patients have been randomly assigned into the following groups: HIIT, MIT, or nonexercise control group (CG). On the third visit (72 to 96 hours after CPX) patients initiated the 16-week exercise program.

**Figure 1:**
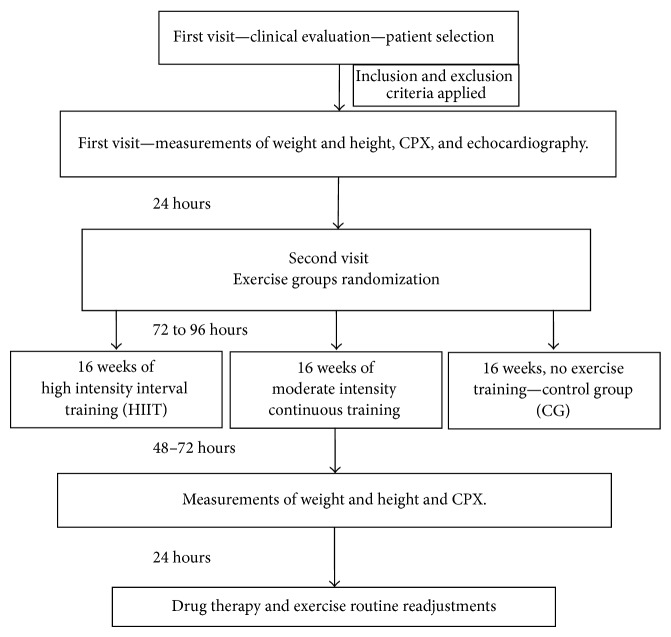
Experimental design.

The CG did not participate in the training program and did not make any kind of regular activity during the 16 weeks of experimental protocol. After completing their planned training program, patients were reevaluated within 48–72 h after the last training session. All patients were sedentary for at least one year prior to the study. Patients in the control group were oriented to maintain their regular habits and not to engage in physical activity programs during the experiment.  Figure 1 exhibits a flow chart of the experimental design.

### 2.3. Cardiopulmonary Exercise Testing (CPX)

Two symptom-limited treadmill (Inbrasport, Porto Alegre, SP, Brazil) running ramp CPX were performed, before and after the exercise intervention. The work rate increments were individualized to elicit each subject's limit of tolerance within 8–12 min, as previously described [21, 22]. Standard criteria for CPX termination were applied, including moderately severe angina, ST depression greater than 2.0 mm, sustained drop in systolic blood pressure, or clinically relevant rhythm disturbance [21]. The Borg 0–10 scale was used to assess the perceived exertion.

Ventilatory assessments were performed via metabolic cart (VO2000, Medical Graphics, Saint Louis, MO, USA). The HR and ventilatory data were analyzed beat-by-beat and averaged every 20 s. The O_2_P was calculated by dividing VO_2_ by HR obtained every 20 s during CPX. Relative O_2_P was calculated by dividing the O_2_P by subject's weight in kilograms. In order to compare the O_2_P curve slopes before and after training it has been assumed that stroke volume responses to exercise would be similar in the rest-exercise transition regardless of the clinical condition. It is well accepted that the stroke volume increases rapidly within the first minute of exercise and this might compromise the linearity of O_2_ pulse at the beginning of exercise. Hence the first minute (rest-exercise transition) of CPX was excluded of the analysis of O_2_P curve in all groups.

Gibbons et al. 2002 [21] said through evidence that the reduction of O_2_P is associated with decreased left ventricular efficiency in the effort. When there was an increase of the absolute values and the inclination, it seems that there was an improvement in left ventricular efficiency [3]. It is shown that the ventricular efficiency can be considered an excellent clinical finding, as the relationship with the survival is direct in CHD.

The OUES was calculated according to recommendations from Baba et al. [23] and Arena et al. [24], using the following equation: VO_2_ = *a* log⁡ VE + *b*, where “*b*” represents the intercept and “*a*” the slope of the curve (OUES). For the calculation of VE/VCO_2_ slopes, data from rest and along exercise were used, as described elsewhere [25].

### 2.4. Echocardiography

The ejection fraction at baseline was determined through echocardiographic images, assessed by a Vivid 7 device (GE Medical Systems, Milwaukee, WI, USA) equipped with a 3.5 MHz transducer. The echocardiogram was performed by a single trained evaluator within a week before the intervention.

### 2.5. Training Program

Patients underwent a supervised treadmill aerobic training, 3 times a week during 16 weeks. The continuous training consisted of 5 min warm-up, followed by 30 min of aerobic training (interval or continuous), and 5 min cool-down. In the continuous training, intensity was constant at 70 to 75% of peak HR. In the interval training sessions, higher (90% peak HR) and lower (60% peak HR) workloads were alternated every 2 min. Once a week, ECG responses to the exercise protocols were checked by a cardiologist, at the beginning and the end of training sessions. Patients were instructed not to enroll in any other exercise program throughout the whole experiment.

### 2.6. Statistical Analyses

Data normality was confirmed by the Kolmogorov-Smirnov test and results are expressed as mean ± SD, unless stated otherwise. Baseline demographic and clinical characteristics among groups were compared by one-way ANOVA and categorical variables were compared by the chi-square test. To compare the results before and after intervention, within and between-group differences were tested by 2-way repeated measures ANOVA followed by Tukey post hoc tests in the event of significant *F* ratios. The relationship between O_2_P and %CPX duration was tested by the Pearson correlation. Subsequently, individual O_2_P slopes were calculated for CPX performed before and after training. Within and between-group differences of O_2_P pattern during CPX, before and after training, were tested by 2-way repeated measures ANOVA, followed by Tukey post hoc verification. All calculations were performed by NCSS statistical software (Kaysville, UT, USA) and statistical significance was set at *P* ≤ 0.05.

## 3. Results

No baseline differences were observed among groups in demographic or medical history data, including age, weight, height, body mass index, ejection fraction, prevalence of risk factors, and interventions. No difference due to gender was detected. Except for the lower prevalence of nitrates in CG, no differences among groups were found for medication use (Table 1). All subjects completed the program, and no untoward events occurred during any of the exercise testing or training procedures. Weight did not change in any group over the study period (*P* = 0.98).

No patient in any group was limited by angina, and none exhibited electrocardiogram evidence of ischemia during baseline CPX. No differences were observed within or between groups in hemodynamic variables such as peak HR, systolic blood pressure (SBP), and diastolic blood pressure (DBP) after the training program (Table 2).

### 3.1. Ventilatory Measurements

Table 2 depicts data for ventilatory variables. After training the peak VO_2_ and peak O_2_P decreased in CG, while increasing in HIIT and remaining stable in MIT. Although data for VE and VO_2_ at ventilatory threshold and peak exercise exhibited similar trends, comparisons among groups did not reach statistical significance. On average, ventilatory threshold was achieved at intensities corresponding to 61% peak VO_2_.

Indices of ventilatory efficiency before and after training are also presented in Table 2. The VE/VCO_2_ slope is maintained in trained groups and in CG. Similar trend was found for OUES.

**Figure 2:**
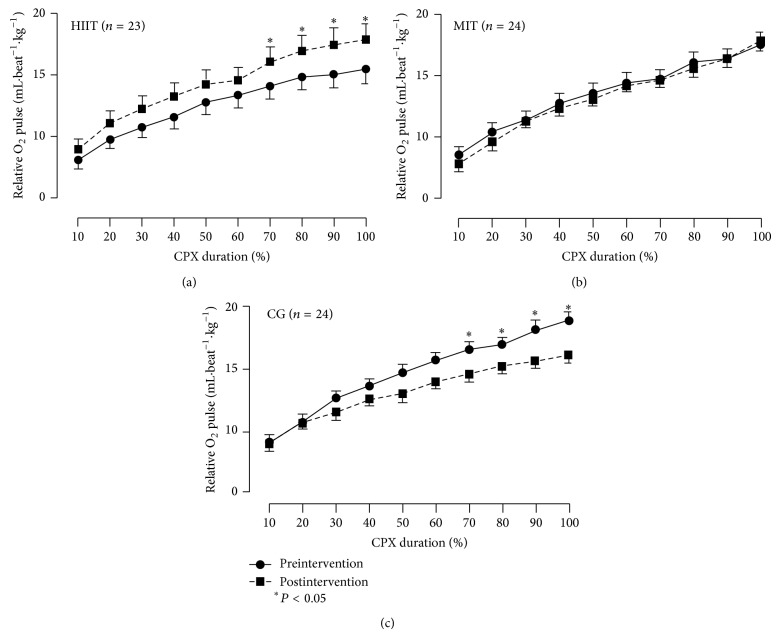
The relative O_2_ pulse curves as a function of percentage time during CPX, before and after intervention. (a) HIIT denotes high intensity interval training group. (b) MIT denotes moderate intensity training group. (c) CG denotes control group. ^*^ *P* < 0.05.

Figure 2 shows the relative O_2_P curves as a function of percentage time during CPX, before and after training. The O_2_P slope increased in HIIT (~22%), remained stable in MIT (~2%), and decreased in CG (~−20%). Differences versus CG were found only in HIIT after 70% of CPX duration (*P* < 0.05)

## 4. Discussion

Three major findings were found in the present study. Firstly, cardiorespiratory fitness and ventricular function (as expressed by peak VO_2_ and O_2_P) in CHD patients were better improved by HIIT compared to MIT. Secondly and most notably, training related differences in O_2_P slope that were observed in HIIT appeared to be greater at higher versus lower exercise intensities (above 70% of CPX duration). Finally, measurements of ventilatory efficiency were not affected by either type of training regimen.

As expected, high intensity exercise training resulted in considerable improvement in peak VO_2_ (18%), which concurs with previous research [11–14]. In contrast, moderate intensity training did not lead to improvements in peak VO_2_, while patients in CG decreased their exercise capacity.

In a recent study, Conraads et al. (2015) [26] demonstrated that HIT and MIT induced substantial and similar increase in VO_2_ peak. However, in that study the mean duration of aerobic training in MIT group was 47 min versus approximately 30 min in our protocol, and CHD patients were 89 versus 24 in the present study. These differences might help explaining the lack of improvement in VO_2_ peak presently observed for MIT.

Taylor et al. (2004) [27] demonstrated through meta-analysis that aerobic training of moderate intensity may not be enough stimulus to increase the VO_2_ peak in subjects with coronary disease—actually overall gains lower than 1 MET have been found. This possibility is consistent with our results, indicating that HIIT, but not MIT was effective to improve the aerobic power in our sample of CHD patients.

To our knowledge, this is the first study comparing the effects of HIIT versus MIT upon O_2_P kinetics during incremental exercise. Similarly to peak VO_2_, only HIIT improved peak O_2_P. This is important, since O_2_P has been considered a surrogate outcome measure for stroke volume during exercise [28]. A flattening response of O_2_P is related to myocardial ischemia in subjects suspected of CHD [3, 29, 30] and higher values are associated with a better prognosis in heart failure patients [31, 32].

Differences in peak O_2_P induced by HIIT were not observed in most submaximal workloads along CPX (Figure 2). Actually, differences in HIIT after training were only observed in intensities above 70% of maximal CPX. A possible explanation for this finding is that diastolic filling time and systolic ejection time progressively decrease when the exercise intensity increases [33]. This reduction may lead to a plateau in stroke volume [3], which is particularly relevant in patients whose ventricular function is impaired [34]. On the other hand, current research suggests that diastolic filling and ventricular emptying are improved in endurance trained subjects, leading to a progressive increase in stroke volume during exercise [35–37]. In brief, an increase in stroke volume during exercise would be due to preserved or enhanced diastolic filling and/or ventricular emptying. Therefore, it is feasible to speculate that only HIIT was able to increase diastolic filling and ventricular emptying at intensities approaching maximal HR, possibly above ventilatory threshold. The present results warrant further investigations comparing the effects of MIT and HIIT upon O_2_P at submaximal workloads, as well as describing its underlying mechanisms in patients with different levels of CHD.

In contrast to the considerable change in exercise capacity following HIIT, modifications in markers of ventilatory efficiency were comparatively modest, regardless of the type of exercise regimen. Although most previous studies have reported improvements in at least some measures of ventilatory efficiency after training, data remain mixed and controversial, with some studies showing improvements in a particular marker, but not in others [6–10]. In the present study, differences due to training in VE/VCO_2_ slope or OUES were not found. These results disagree with previous research with heart failure patients [5, 19].

There are several potential explanations as to why a significant change in VE/VCO_2_ slope and OUES did not occur in the current study. Firstly, all heart failure subjects were excluded from the study, remaining therefore comparatively healthy patients. In addition, all patients were stable at the time of enrollment and with a mean ejection fraction of approximately 63% at baseline. The mean VE/VCO_2_ slope and OUES at baseline for all subjects (27 and 1.9, resp.) were below the usual threshold for elevated risk (typically above 34 for VE/VCO_2_ slope and below 1.4 for OUES) and below values adopted by studies reporting an improvement in these markers due to exercise training [7, 19, 38, 39]. Therefore, it is likely to think that our patients had less room to exhibit gains in ventilatory efficiency than patients with more severe disease included in previous studies.

The present study has limitations. As mentioned earlier, our sample was composed by patients with mild or moderate severity of CHD, with a mean ejection fraction of 63%, which may have affected the influence of training on ventilatory efficiency. A precise determination of the mechanisms underlying the effects of exercise training on ventilatory efficiency and stroke volume would require more invasive measures of lung perfusion, arterial blood gases, and ventricular function, which were not available in the present study.

## 5. Conclusions

Peak VO_2_, peak O_2_P, and O_2_P curve pattern in patients with CHD were improved by HIIT, but not by MIT. On the other hand, markers of ventilatory efficiency were not influenced by any type of exercise training. Further studies are needed to determine the underlying mechanisms associated with exercise-related improvement in O_2_P, as well as to investigate the effects of different exercise regimens upon the ventilatory efficiency of patients with different degrees of cardiac disease.

## Figures and Tables

**Table 1:** Demographic and clinical characteristics among groups.

Variables	CG (*n* = 24)	MIT (*n* = 24)	HIT (*n* = 23)	*P* value^**^
Demographic characteristics				
Age (yrs)	64 ± 12	62 ± 12	56 ± 12	0.07
Weight (Kg)	76 ± 13	74 ± 15	78 ± 19	0.73
Height (cm)	169 ± 9	167 ± 6	169 ± 9	0.66
Body mass index (Kg/m^2^)	26.9 ± 4.4	26.8 ± 4.8	27.5 ± 5.9	0.89
Male (%)	76	66	63	0.73
Ejection fraction (%)	67 ± 10	60 ± 14	63 ± 12	0.16
Medications *n* (%)				
Beta-blocker	83	92	78	0.43
Diuretic	54	50	35	0.37
Angiotensin converting enzyme inhibitor	17	42	26	0.15
Antialdosterone	12	21	17	0.74
Statin	87	83	83	0.88
Calcium channel blockers	8	0	4	0.35
Nitrate	21	62^*^	52^*^	0.01
Medical history *n* (%)				
Diabetes	25	25	30	0.88
Hypertension	75	67	61	0.58
Smoking	17	17	13	0.88
Dyslipidemia	67	58	52	0.59
Myocardial infarction	62	62	43	0.31
Ischemic heart disease	54	58	43	0.57
Interventions *n* (%)				
Percutaneous transluminal coronary angioplasty, stenting or both	96	92	83	0.3
Myocardial revascularization	83	67	65	0.3

Plus-minus values are means ± SD. HIIT denotes high intensity interval training.

MIT denotes moderate intensity training. CG denotes control group.

^*^Denotes differences from control group (*P* < 0.05).

^**^ *P* values of the ANOVA.

**Table 2:** Ventilatory results of CPX at peak and ventilatory threshold among groups.

Variable	CG (*n* = 24)		MIT (*n* = 24)		HIIT (*n* = 23)		*P*
	Before	After	Before	After	Before	After	Interaction
Peak CPX results							
VE (L/min)	48 ± 15	42 ± 16	43 ± 8	46 ± 15	47 ± 13	55 ± 16	0.09
VO_2_ peak (mL·Kg^−1^·min^−1^)	21.9 ± 6	18.6 ± 6^∗†^	21.8 ± 6	21.9 ± 6	20.6 ± 5	24.4 ± 5^*^	0.04
Oxygen pulse (mL/beat)	13.7 ± 4	11.7 ± 4^∗†^	12.5 ± 4	12.7 ± 4	12.4 ± 4	14.2 ± 4^*^	0.05
VE/VCO_2_ slope	27.4 ± 3.9	28.1 ± 3.2	27.9 ± 4.6	26.8 ± 3.3	27.6 ± 4.0	27.3 ± 4.1	0.48
OUES	1.9 ± 0.6	1.7 ± 0.6	1.9 ± 0.5	1.8 ± 0.5	1.8 ± 0.6	2.0 ± 0.6	0.16
RER	1.03 ± 0.1	1.02 ± 0.1	1.02 ± 0.1	1.01 ± 0.1	1.05 ± 0.1	1.07 ± 0.1	0.53
HR (bpm)	122 ± 26	122 ± 28	127 ± 18	128 ± 19	131 ± 25	133 ± 24	0.99
SBP (mmHg)	181 ± 26	170 ± 25	172 ± 41	157 ± 53	173 ± 21	169 ± 23	0.7
DBP (mmHg)	77 ± 8	73 ± 9	74 ± 20	66 ± 22	74 ± 6	69 ± 9	0.72
Perceived exertion	19 ± 1.3	18 ± 1.5	17 ± 2	18 ± 1.7	18 ± 1.7	19 ± 1.5	0.56
Ventilatory threshold							
VE (L/min)	24.2 ± 8	20.8 ± 6	21.0 ± 6.5	20.4 ± 4.8	22.6 ± 5.2	25.2 ± 6.1	0.06
VO_2_ (mL·Kg^−1^·min^−1^)	15.0 ± 3.7	13.1 ± 3.7	13.8 ± 3.6	13.4 ± 2.7	14.2 ± 3.8	15.6 ± 4.6	0.09
Oxygen pulse (mL/beat)	11.7 ± 3.3	10.4 ± 3.0	10.5 ± 3.3	10.1 ± 2.2	11.0 ± 3.4	11.9 ± 3.3	0.21

Plus-minus values are means ± SD. ^*^Denotes *P* < 0.05 for within group comparison (pre versus post training).

CPX denotes cardiopulmonary exercise testing.

HIIT denotes high intensity interval training. MIT denotes moderate intensity training.

CG denotes control group. VE denotes ventilation.

OUES denotes oxygen uptake efficiency slope. RER denotes respiratory exchange ratio.

HR denotes heart rate. SBP denotes systolic blood pressure. DBP denotes diastolic blood pressure.

^†^Denotes *P* < 0.05 for CG versus HIIT.
